# The *Drosophila* SH2B Family Adaptor Lnk Acts in Parallel to Chico in the Insulin Signaling Pathway

**DOI:** 10.1371/journal.pgen.1000596

**Published:** 2009-08-14

**Authors:** Christian Werz, Katja Köhler, Ernst Hafen, Hugo Stocker

**Affiliations:** 1 Institute of Molecular Systems Biology, Zurich, Switzerland; 2 PhD Program for Molecular Life Sciences, Life Science Zurich Graduate School, Zurich, Switzerland; University of California San Francisco, United States of America

## Abstract

Insulin/insulin-like growth factor signaling (IIS) plays a pivotal role in the regulation of growth at the cellular and the organismal level during animal development. Flies with impaired IIS are developmentally delayed and small due to fewer and smaller cells. In the search for new growth-promoting genes, we identified mutations in the gene encoding Lnk, the single fly member of the SH2B family of adaptor molecules. Flies lacking *lnk* function are viable but severely reduced in size. Furthermore, *lnk* mutants display phenotypes reminiscent of reduced IIS, such as developmental delay, female sterility, and accumulation of lipids. Genetic epistasis analysis places *lnk* downstream of the *insulin receptor* (*InR*) and upstream of *phosphoinositide 3-kinase* (*PI3K*) in the IIS cascade, at the same level as *chico* (encoding the single fly insulin receptor substrate [IRS] homolog). Both *chico* and *lnk* mutant larvae display a similar reduction in IIS activity as judged by the localization of a PIP_3_ reporter and the phosphorylation of protein kinase B (PKB). Furthermore, *chico*; *lnk* double mutants are synthetically lethal, suggesting that Chico and Lnk fulfill independent but partially redundant functions in the activation of PI3K upon InR stimulation.

## Author Summary

The regulation of growth is among the most fundamental processes during development of multicellular organisms. Research over the past years has established a key function of the insulin/insulin-like growth factor signaling (IIS) pathway in ensuring proper growth at the cellular and the organismal level. Impaired IIS has been associated with diseases such as type 2 diabetes, leprechaunism, and heart disease; and deregulated IIS often contributes to the development of cancer. Here, we describe the characterization of the *Drosophila* SH2B family adaptor protein Lnk. Mutants in *lnk* are viable but unable to reach the normal size due to a reduction in cell size and cell number. Our characterization of *lnk* mutant flies has revealed phenotypes associated with impaired IIS, such as developmental delay, female sterility, and increased lipid levels in adults. Using a combination of genetic interaction experiments and molecular readouts for IIS activity, we demonstrate that Lnk acts in parallel to the IRS homolog Chico downstream of the insulin receptor to regulate cellular growth.

## Introduction

The control of cell, organ and body size is tightly regulated to ensure proper development of multicellular organisms. A key pathway controlling growth, metabolism, reproduction and longevity is the insulin/insulin-like growth factor signaling (IIS) pathway [1]. The insulin receptor (InR) and the corresponding downstream core components are conserved in *Drosophila* [2]–[4], mediating cell growth and cell division in response to environmental factors such as nutrient availability through a series of protein-protein interactions and phosphorylation events [5].

The core components of the *Drosophila* IIS pathway include Chico, the homolog of the insulin receptor substrates (IRS), the lipid kinase phosphoinositide 3-kinase (PI3K), the lipid phosphatase PTEN, and the serine-threonine kinase PKB [6]. Chico gets phosphorylated upon IIS pathway activation, providing binding sites for the Src Homology 2 (SH2) domain of p60, the regulatory subunit of PI3K. Increased PI3K activity leads to the accumulation of phosphatidylinositol-(3,4,5)-trisphosphate (PIP_3_) at the plasma membrane, which recruits PKB to the membrane via its pleckstrin homology (PH) domain. PKB takes a central position in the regulation of multiple cellular processes such as cellular growth, proliferation, apoptosis, transcription and cell motility [7].

In *Drosophila*, mutations in IIS components result in reduced cell, organ and body size with little effect on cell fate and differentiation. For example, hypomorphic mutants of essential IIS components and, in particular, homozygous null mutants of *chico* are viable but only approximately half the size of wild-type flies, due to smaller and fewer cells. Furthermore, characteristic defects caused by reduced IIS activity include female sterility, an increase in total lipid levels of adults, and a severe developmental delay [8],[9].

*chico* encodes an adaptor protein, a group of proteins without catalytic activity usually carrying domains mediating specific interactions with other proteins such as an SH2 domain, a PH domain, or a phosphotyrosine-binding (PTB) domain. Adaptor proteins play an important role in the formation of protein-protein interactions and thus in the formation of protein networks. The various interaction domains within adaptor proteins and the specificity of those domains provide adaptor molecules with the ability to elicit characteristic responses to a particular signal.

Recently, a novel family of adaptor proteins, the SH2B family, has been identified in mammals. It consists of three members – SH2B1 (SH2B/PSM), SH2B2 (APS) and SH2B3 (Lnk) – that share a common protein structure with an N-terminal proline-rich stretch, a PH domain, an SH2 domain and a highly conserved C-terminal Cbl recognition motif [10]–[12]. They have been shown to regulate signal transduction by receptor tyrosine kinases such as the InR, IGF-I receptor and receptors for nerve growth factor, hepatocyte growth factor, platelet-derived growth factor and fibroblast growth factor, as well as by the JAK family of tyrosine kinases [11], [13]–[17]. Whereas SH2B3 (Lnk) has been described to function exclusively by negatively regulating receptor kinases that are specialized in the development of a subset of immune and hematopoietic cells, the picture for the other two family members is not as clear yet [18].

Although both SH2B1 and SH2B2 have been shown to be directly involved in the regulation of JAK tyrosine kinases and of IIS, their specificities and physiological functions are complex and remain largely elusive. For example, depletion of SH2B1 in mice leads to severe obesity, leptin and insulin resistance as well as female infertility [19],[20]. However, a number of studies suggest that SH2B1 exerts its function predominantly in the association with JAK2 and regulation of related signaling cascades [21]. For example, binding of SH2B1 to JAK2 results in an enhancement of JAK2 activation and JAK2-mediated growth hormone signaling [22], and depletion of SH2B1 leads to decreased leptin-stimulated JAK2 activation and reduced phosphorylation of its substrates [19].

SH2B2 is also able to bind to JAK2 and to the InR [13],[23] but recent research has mainly focused on the mechanisms related to the connection of SH2B2 and c-Cbl [13],[24],[25]. Phosphorylation of Tyr618 in SH2B2 stimulates binding of c-Cbl and thus mediates GLUT4 translocation and inhibition of erythropoietin-dependent activation of Stat5 [13],[24]. However, the general impact of SH2B2 on receptor tyrosine kinase signaling remains controversial. Whereas Ahmed and colleagues showed that SH2B2 overexpression delayed InR and IRS dephosphorylation and enhanced PKB activation [25], several other studies (e.g. on SH2B2 knockout mice) have suggested a negative regulatory role for SH2B2 in IIS, which might also be mediated via c-Cbl dependent ubiquitination and subsequent degradation of target kinases [26],[27].

Although interactions with the IIS pathway and the InR have been described for SH2B1 and SH2B2, the physiological significance of these connections in mammals appears to be the regulation of metabolism and energy homeostasis rather than the control of cell growth and proliferation [19],[28].

In contrast to the mammalian situation, the *Drosophila* genome encodes a single adaptor protein that shares a common domain structure with the SH2B family, termed Lnk. Here, we show that *Drosophila lnk* predominantly regulates cellular and organismal growth in a cell-autonomous way. We observed that loss of *lnk* function leads to a reduction in cell size and cell number, reminiscent of decreased IIS activity. A thorough genetic analysis placed Lnk as a positive regulator of IIS at the level of IRS/Chico.

## Results/Discussion

### *Drosophila lnk* Regulates Growth and Body Size

**Figure 1:**
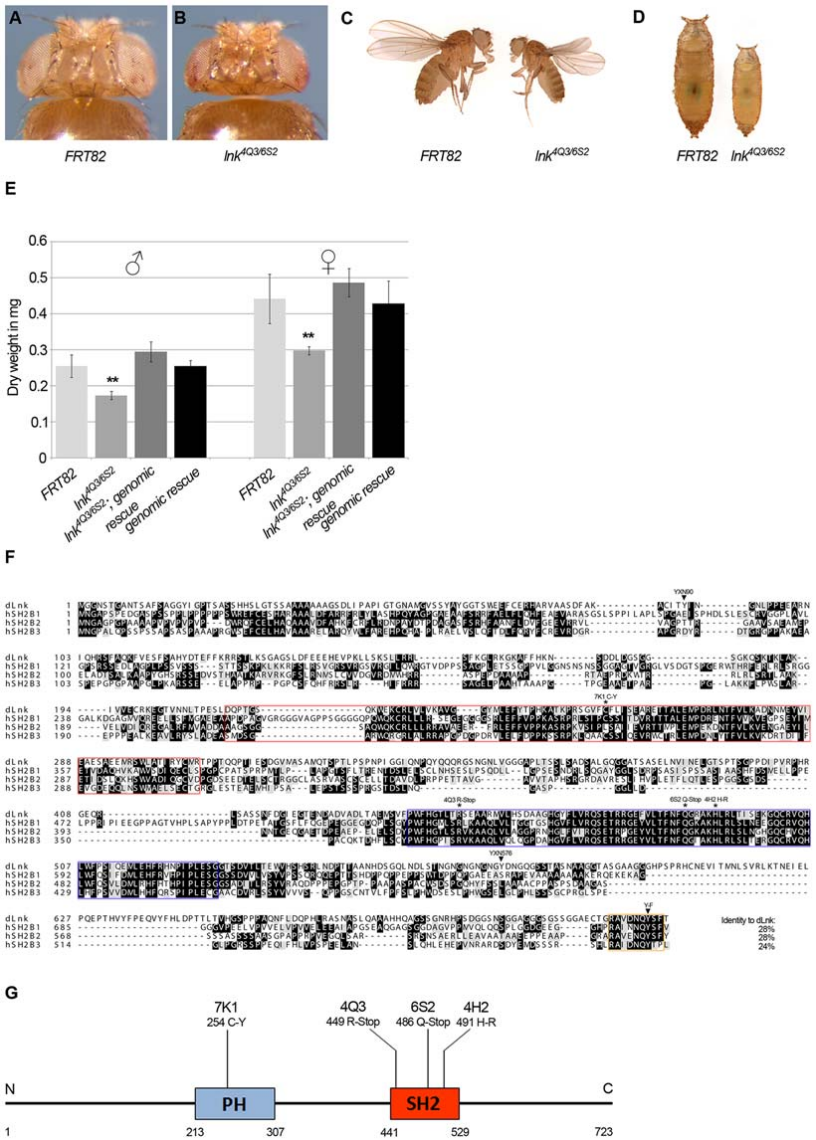
Flies mutant for *lnk* are viable but small. (A–D) *lnk* regulates organismal size throughout development. In comparison to *ey-flp* induced control clones (*FRT82*, A), *FRT82 lnk^4Q3^* clones result in a small head phenotype (B). *lnk^4Q3^*/*lnk^4Q3^* adult flies (C) and pupae (D) are smaller than the controls. (E) Flies lacking *lnk* function are strongly reduced in dry weight. Introduction of a genomic construct comprising the *lnk* locus rescues the *lnk* growth deficit. Significant changes relative to the control (*p*≤0.01, Student's t-test, n = 20) are marked by double asterisks; error bars represent the standard deviation. (F) Alignment of the *Drosophila* Lnk protein and its human homologs of the SH2B family of adaptor proteins. *lnk* codes for a 723 amino acid adaptor protein containing a PH domain and an SH2 domain. Black and grey boxes indicate amino acid identity and similarity, respectively. The SH2 domain is highlighted in red, the PH domain in blue, and the highly conserved Cbl binding motif in orange. Asterisks mark the mutations recovered in the screen, and arrowheads indicate the tyrosines of the two potential YXN Drk SH2 binding motifs and the conserved Cbl binding motif. (G) The mutations leading to an amino acid exchange in the PH domain (7K1) and to premature translational stops (4Q3, 6S2) or an amino acid exchange in the SH2 domain (4H2), respectively, genetically behave as null alleles, indicating that both the PH and the SH2 domain are essential for Lnk's function. Genotypes are: (A) *y w ey-flp*/*y w*; *FRT82*/*FRT82 cl(3R3) w^+^*, (B) *y w ey-flp*/*y w*; *FRT82 lnk^4Q3^*/*FRT82 cl(3R3) w^+^*.

We identified *lnk* in an unbiased screen for growth-regulating genes based on the eyFLP/FRT technique in *Drosophila*. In principle, mutations in growth-promoting genes led to flies with smaller heads (the so-called pinheads), whereas negative regulators of tissue growth resulted in larger heads (referred to as bighead mutants). Among others, we identified four mutations causing a pinhead phenotype that fell into a single complementation group on the right arm of the third chromosome (Figure 1B). We mapped the complementation group close to the *lnk* locus (CG 17367) at the cytological position 96F. Subsequent sequencing revealed EMS-induced mutations in the *lnk* coding region for each allele.

Flies homozygous mutant for *lnk* are small but do not show any obvious patterning defects (Figure 1C). Homozygous mutant pupae are also small, indicating that *lnk* is essential for proper organismal growth throughout development (Figure 1D). *lnk* mutant flies are severely reduced in dry weight, as shown for male and female flies (Figure 1E). This defect is fully rescued by introducing a genomic rescue construct comprising the entire *lnk* locus, proving that the mutations in *lnk* are responsible for the growth phenotype (Figure 1E).

The most closely related group of proteins to *Drosophila* Lnk in vertebrates is the SH2B family of adaptor proteins sharing a common protein structure. Alignment of *Drosophila* Lnk with its human homologs (SH2B1, SH2B2 and SH2B3) shows high sequence identity in particular in the conserved PH and SH2 domains (Figure 1F, Figure S2). The four *lnk* alleles recovered in the screen (7K1, 4Q3, 6S2, 4H2) contain a single point mutation in either of these two highly conserved protein domains resulting in a premature stop (4Q3, 6S2) or an amino acid exchange in conserved residues (7K1, 4H2) (Figure 1F and 1G). Since hemizygous and heteroallelic *lnk* mutant animals display identical phenotypes, all *lnk* alleles are genetically null, suggesting an essential role of both the PH and the SH2 domain for Lnk function.

### Lnk Is a Component of the IIS Pathway

SH2B1 and SH2B2, two members of the mammalian family of Lnk-related adaptor proteins, have been shown to associate with several signaling molecules including JAK2 and the InR [11],[21],[29]. However, the different proteins seem to have distinct impacts on the respective pathways, regulating them either in a positive or negative manner [29],[30]. Using the new mutations in the single member of the SH2B family in *Drosophila* allowed us to determine whether *lnk* plays an essential role in either of these pathways.

Although the tyrosines in JAK2 and JAK3 mediating their interaction with the SH2B family proteins in mammals are not conserved in the *Drosophila* homolog, we wondered whether Lnk has a function in the regulation of *Drosophila* JAK. Misregulation of JAK/Stat signaling in *Drosophila* results in formation of melanotic tumors and proliferative defects in larval blood cells, held out wings and rough or disrupted eye phenotypes as well as male sterility and fused egg chambers in the vitellarium due to the absence of stalk cells [31]–[34]. In our characterization of homozygous *lnk* mutant animals we did not observe any of the phenotypes that are characteristic for impaired JAK/Stat signaling (data not shown). Moreover, genetic interaction experiments of *lnk* with any of the core JAK/Stat pathway components did not reveal a connection of Lnk to JAK/Stat signaling. These results suggest that in *Drosophila*, Lnk is not involved in the regulation of signaling activity downstream of JAK.

**Figure 2:**
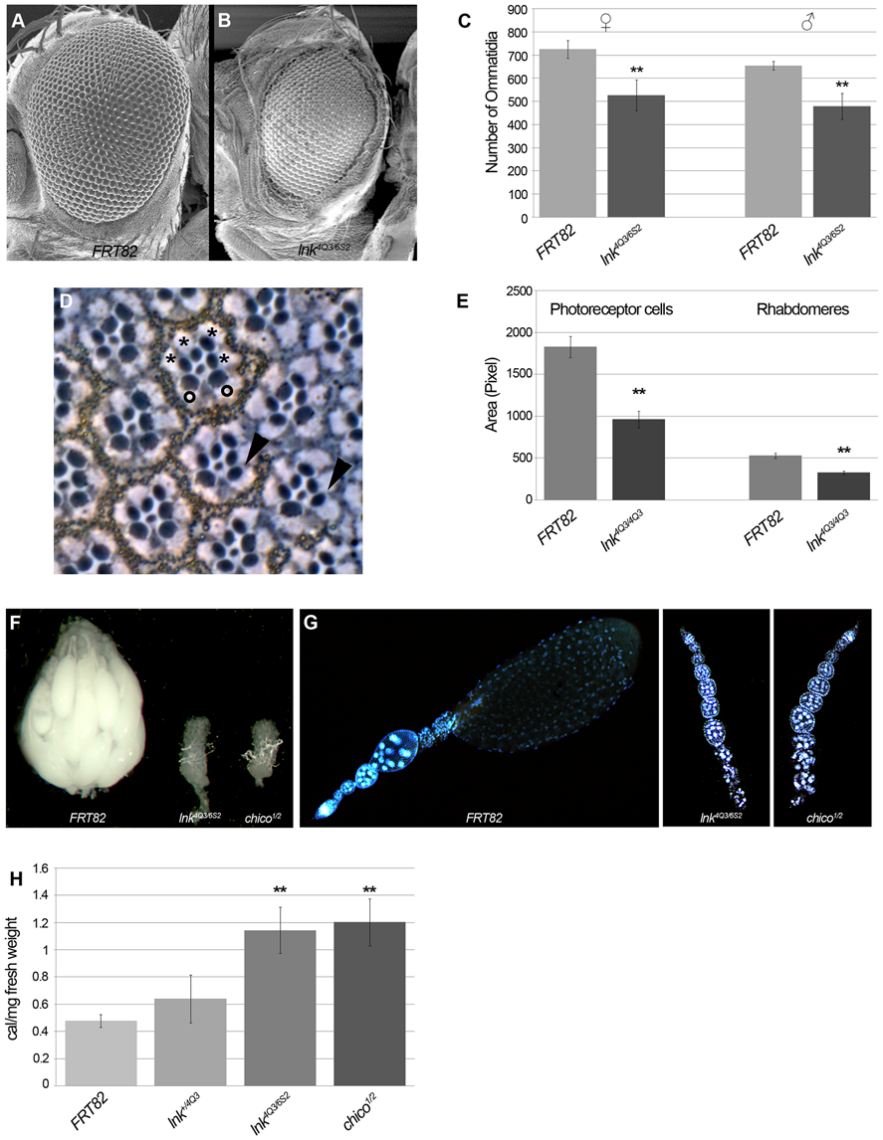
*Drosophila lnk* regulates cell number and cell size, reminiscent of low IIS activity. SEM picture of a wild-type *Drosophila* head (A) compared to the head of a homozygous *lnk* mutant fly (B). The eye of the *lnk* mutant is smaller due to fewer and smaller ommatidia. (C) Whereas wild-type eyes consist of more than 700 ommatidia, the number of ommatidia is reduced to about 500 in *lnk* mutants. (D) Tangential sections through mosaic eyes consisting of homozygous mutant photoreceptors (marked by the absence of pigment) surrounded by heterozygous tissue. Ommatidia containing both wild-type and small homozygous *lnk* mutant photoreceptor cells (arrowheads) can be observed, pointing to a cell autonomous role of *lnk* in cell size regulation (wild-type cells are marked by circles and mutant cells by asterisks in a representative ommatidium). (E) The sizes of photoreceptor cells and of rhabdomeres are reduced in *lnk* mutant ommatidia compared to wild type. (F) *lnk* mutant females are sterile due to an arrest in oogenesis. Ovaries of homozygous *lnk* females are small and contain only few oocytes developed to previtellogenic stages (G). The ovarioles of *lnk* mutant females resemble those of *chico* mutants (G). (H) The lipid levels of *lnk* mutant males are strongly elevated compared to wild type, similar to the levels measured in *chico* mutant flies. Significant changes relative to the respective controls (*p*≤0.01, Student's t-test) are marked by double asterisks; error bars represent the standard deviation; n = 8 in (C), n = 9 in (E), n = 10 in (H).

The initial observation that *lnk* mutations reduced organ and body size pointed at a role of Lnk in the IIS pathway. We characterized the growth phenotype of *lnk* mutants further by quantifying ommatidia number and generating tangential sections of mosaic eyes to study the impact of *lnk* on cell number and cell size (Figure 2A–2E). SEM pictures of heads of *lnk* mutant adults compared to wild type and quantification of ommatidia number revealed that mutations in *lnk* caused a reduction in cell number by about 30% (Figure 2A–2C). Induction of *lnk* mutant clones in the eye resulted in a cell-autonomous reduction of cell size in photoreceptor cells and rhabdomeres, as shown by tangential eye sections (Figure 2D) and subsequent quantification of photoreceptor cell and rhabdomere area in *lnk* mutant tissue compared to wild type (Figure 2E). Therefore, *lnk* function is important to ensure proper regulation of cell number and cell size, similar to IIS components.

It has previously been shown that IIS is required in oogenesis beyond the last previtellogenic stage; a reduction in IIS activity leads to an arrest in oogenesis and female sterility [35]. Female flies lacking *lnk* function are also sterile and have small ovaries. These ovaries only contain oocytes that developed until the last previtellogenic stage and resemble ovaries of females mutant for *chico* (Figure 2F and 2G).

A further characteristic phenotype of impaired IIS is the accumulation of lipids in adult flies. The lipid levels in three-day old male *chico* flies are more than twice the level than in the control despite their smaller body size [8]. Homozygous *lnk* mutant flies reach the same lipid levels as *chico* mutants (Figure 2H). Taken together, these results strongly indicate a role of Lnk in the IIS pathway.

The phenotypes of homozygous *lnk* mutants suggest that Lnk regulates cellular growth exclusively via IIS. However, the protein sequence of Lnk contains two putative Drk/Grb2 YXN binding sites (Figure 1F). In addition, all SH2B family members, except for the beta, gamma and delta isoform of SH2B1, carry a highly conserved consensus site for binding of Cbl [36]. The functionality of this Cbl binding site has only been demonstrated in SH2B2 so far [24],[25]. In order to test the functional significance of the individual binding motifs, we generated rescue constructs consisting of the genomic *lnk* locus but carrying specific mutations that result in amino acid exchanges in the core tyrosine of the respective motifs. These constructs fully rescued the reduction in dry weight in *lnk* mutants, suggesting that neither binding of Drk to the YXN site nor an interaction of Lnk with Cbl through the C-terminal binding motif is important in the regulation of growth (Figure S1A, S1B, S1C). In contrast, both the PH and the SH2 domains of Lnk are essential for its function because the *lnk* alleles disrupting either domain behave genetically as null mutations.

**Figure 3:**
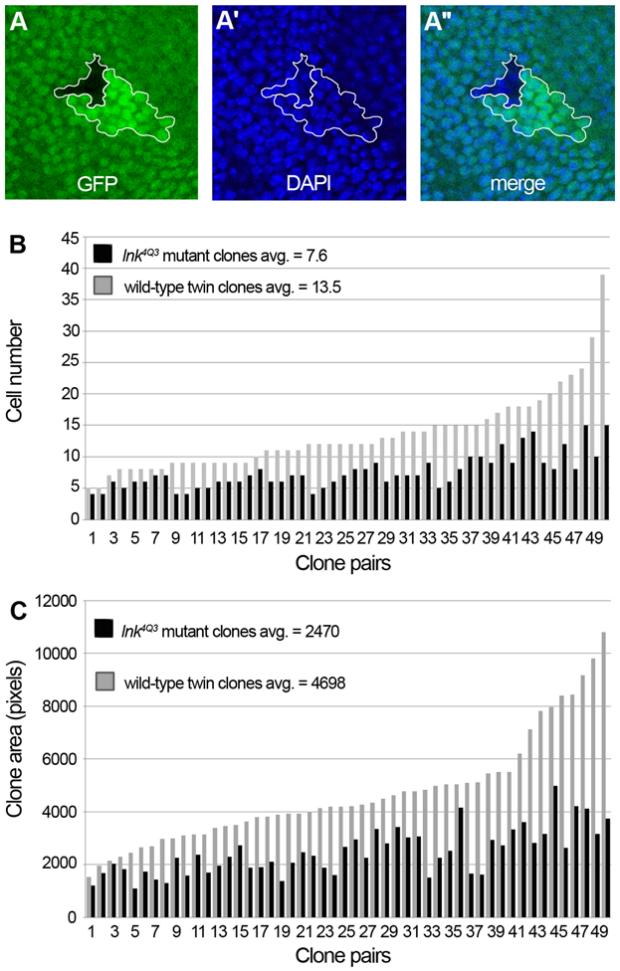
Clones of *lnk* mutant cells are smaller due to fewer and smaller cells. (A) Twin-spot clone in the wing imaginal disc. A clone consisting of *lnk* mutant cells (marked by the absence of GFP) and its wild-type sister clone (marked by two copies of GFP) were induced by mitotic recombination using the hsFlp/FRT system. Nuclei are stained with DAPI (A′) and merged with the GFP signal (A″). Clones of *lnk* mutant cells consist of fewer cells (B) and cover smaller areas (C) than their corresponding wild-type sister clones, indicating that *lnk* is required for proper cellular growth during development.

In order to study the consequences of the loss of *lnk* function on cell growth, we performed a clonal analysis in larval wing discs using the *4Q3* allele. We used the hsFLP/FRT system to induce mitotic recombination, thus to generate homozygous *lnk* mutant cell clones (marked by the absence of GFP) adjacent to clones that consist of wild-type cells (marked by two copies of GFP) (Figure 3A). All mutant clones were smaller than their wild-type sister clones (Figure 3C), and they contained fewer cells (Figure 3B). Although a clear tendency to a cell size reduction of *lnk* mutant cells, as determined by the ratio of clone area to cell number, was apparent, the relative reduction was not significant in larval wing discs. We thus speculate that the influence of *lnk* on cell size is rather subtle in early stages of development.

**Figure 4:**
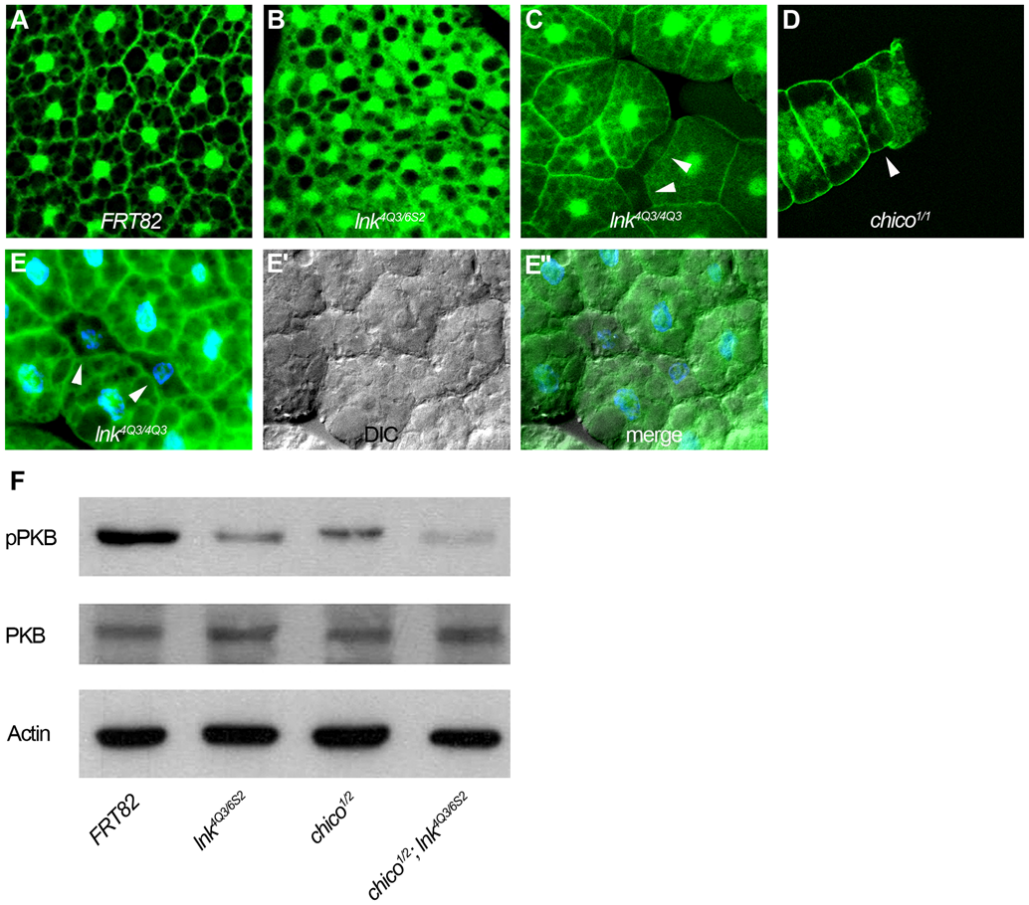
*lnk* affects IIS activity. (A–D) The IIS activity is visualized by the localization of the tGPH reporter. Compared to the signal at the membrane of wild-type fat body cells (A), the signal is diffuse and mostly cytoplasmic in *lnk* mutant larvae (B), indicative of low PI3K activity. In clones of *lnk^4Q3^* mutant cells (recognizable based on the size reduction and indicated by arrowheads), the signal is also almost absent from the plasma membrane (C). A similar effect is observed in *chico* mutant cells (D). (E–E″) The strong reduction of membrane-localized tGPH is not due to structural defects of the *lnk* mutant cells as shown by differential interference contrast (DIC) microscopy (E′–E″). (F) The phosphorylation of PKB is used to monitor IIS activity in larval extracts. Both *lnk* and *chico* mutants display a clear reduction of phosphorylated PKB. Note that the levels of PKB do not change. Actin is used as a loading control.

We further used molecular readouts of IIS activity to investigate the consequences of the loss of *lnk* function. Stimulation of the InR activates PI3K, which increases the levels of phosphatidylinositol-(3,4,5)-trisphosphate (PIP_3_) at the plasma membrane [6]. Previously, a reporter containing a PH domain fused to GFP (tGPH) that localizes to the plasma membrane as a result of PI3K activity had been described [37]. Using this reporter, we monitored PIP_3_ levels in wild-type and *lnk* mutant fat body cells as well as in clones of *lnk* mutant cells in the fat body. Whereas the tGPH reporter localized to the membrane in wild-type cells (Figure 4A), the GFP signal was predominantly observed in the cytoplasm in *lnk* mutant cells (Figure 4B and 4C), indicating that the loss of *lnk* function causes a reduction of PI3K signaling activity. The impact of *lnk* on tGPH localization is comparable to the effects observed in *chico* mutant cells (Figure 4D).

As another molecular readout of IIS activity, we measured the phosphorylation levels of PKB, a downstream kinase of IIS. Lysates of homozygous *lnk* and *chico* mutant larvae were subjected to Western analysis and compared to wild-type controls. Whereas the PKB protein levels were comparable in all genotypes, the amount of phosphorylated PKB was reduced in both *lnk* and *chico* mutant larvae (Figure 4F). Thus, Lnk and Chico contribute similarly to the activity of PI3K.

### Lnk Acts Downstream of the InR in Parallel to Chico

**Figure 5:**
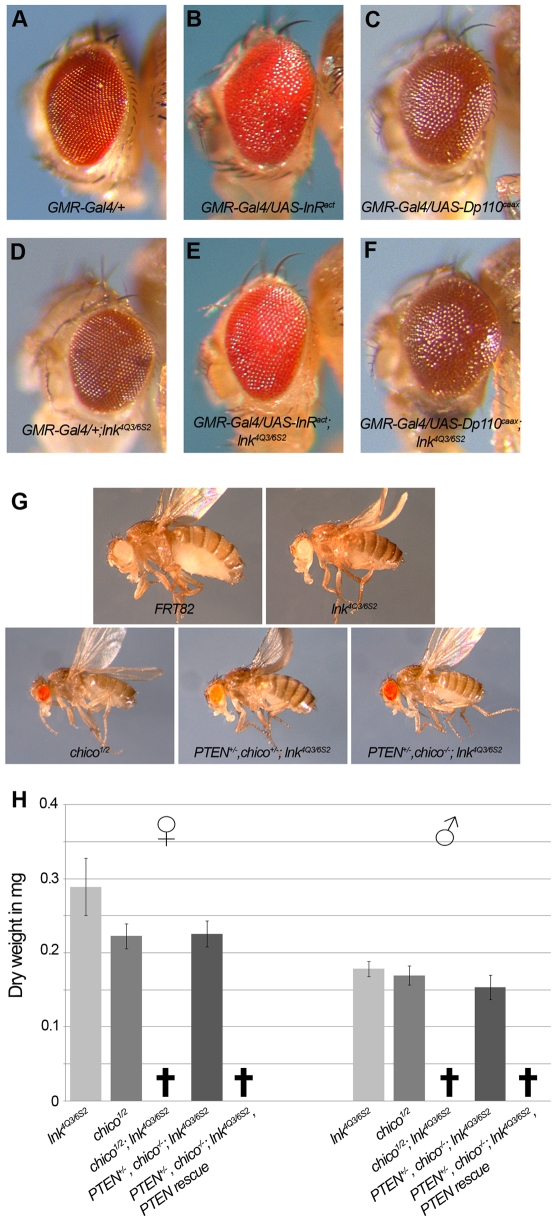
*lnk* genetically interacts with components of the IIS pathway. (A–F) Whereas the loss of *lnk* function suppresses the overgrowth phenotype caused by eye-specific expression of a constitutive active form of InR (compare E with B), it is not sufficient to suppress the overgrowth phenotype caused by an activated form of PI3K (compare F with C). (G,H) *chico* and *lnk* display synthetic lethality. Removing one copy of *PTEN* in a *chico*; *lnk* mutant background is sufficient to restore viability of *chico*; *lnk* double mutant flies. Re-introduction of a *PTEN* genomic rescue construct into this background results in lethality. Error bars represent the standard deviation; n = 20. Genotypes are: (A) *GMR-Gal4*/+, (B) *GMR-Gal4*/*UAS-InR^act^*, (C) *GMR-Gal4*/*UAS-Dp110^CAAX^*, (D) *GMR-Gal4*/+; *lnk^4Q3^*/*lnk^6S2^*, (E) *GMR-Gal4*/*UAS-InR^act^*; *lnk^4Q3^*/*lnk^6S2^*, (F) *GMR-Gal4*/*UAS-Dp110^CAAX^*; *lnk^4Q3^*/*lnk^6S2^*, (G) *FRT82*, *lnk^4Q3/6S2^*, *chico^1/2^*, *Df(2L)Exel6026*/+; *lnk^4Q3^*/*lnk^6S2^* and *chico^1^*/*Df(2L)Exel6026*; *lnk^4Q3^*/*lnk^6S2^*.

In order to establish where *lnk* acts in the IIS cascade, we performed genetic epistasis experiments. We tested the ability of *lnk* to suppress the overgrowth phenotype caused by overexpression of InR during eye development (Figure 5B). In this sensitized background loss of *lnk* function reduced the eye size almost to wild-type size, suggesting that Lnk modulates the IIS pathway downstream of the receptor (Figure 5E). In contrast, homozygosity for *lnk* was not sufficient to suppress the overgrowth caused by a membrane-tethered form of PI3K (Figure 5C and 5F). Thus, Lnk acts between the InR and the lipid kinase PI3K in the IIS pathway.

The phenotypic similarities between *lnk* and *chico* mutants are striking. Both genes encode adaptor proteins with a PH domain and a phosphotyrosine-binding motif (an SH2 domain in the case of Lnk and a PTB domain in the case of Chico, respectively), and both act between the InR and PI3K. Thus, it is conceivable that Lnk is required for proper Chico function, for example by stabilizing the phosphorylated InR and thereby allowing a stable InR-Chico interaction. We attempted to genetically test whether Lnk acts via Chico. If this were the case, *chico*; *lnk* double mutants would be expected to display similar phenotypes as the single mutants. However, *chico*; *lnk* double mutants were lethal (Figure 5H). Removing one copy of *PTEN* (encoding the lipid phosphatase that antagonizes PI3K) restored viability of the *chico*; *lnk* double mutants (Figure 5G and 5H), suggesting that the *chico*; *lnk* double mutants suffer from reduced IIS activity and thus insufficient levels of the second messenger PIP_3_. Reducing the amount of PTEN, the negative regulator of PIP_3_ production, allows for PIP_3_ levels above a critical threshold for survival but still insufficient to ensure normal growth. These results imply that Chico and Lnk independently act downstream of the InR, and that both adaptors are required for the full activation of PI3K upon InR stimulation. Consistently, we found that the levels of phospho-PKB were further reduced in *chico*; *lnk* double mutant larvae as compared to single mutants (Figure 4F).

Our data clearly indicate that both Lnk and Chico are required for the full activity of PI3K, with each adaptor being sufficient for a partial stimulation of PI3K activity. This might explain why *chico* and *lnk* are among the few non-essential genes in the IIS cascade. How does Lnk contribute to the activation of PI3K? Probably, Lnk does not exert its function in the same way as Chico. In contrast to Chico, Lnk lacks an YXXM consensus binding site for the SH2 domain of the regulatory subunit of PI3K. Upon activation of the InR, Lnk might connect the signal from the InR with Chico in order to enhance PI3K activation. Interestingly, such a mechanism has been proposed in vertebrates, where SH2B1 promotes IRS1 and IRS2-mediated activation of the PI3K pathway in response to Leptin [38]. However, we favor a model in which Lnk promotes the membrane localization of PI3K by recruiting another binding partner of PI3K or by counteracting a negative regulator of PI3K localization. It will thus be important to identify physical interactors of Lnk.

## Materials and Methods

### Fly Stocks

Four EMS induced *lnk* alleles on *FRT82B* chromosomes were recovered in a mosaic screen based on the eyFLP/FRT cell lethal technique [39]. The complementation group was mapped close to an *y^+^* marked transgene in 96E, and the map position was refined to 96F by non-complementation with *Df(3R)Espl3* (96F1; 97B1, Bloomington stock number 5601) and complementation with *Df(3R)ME61* (96F12-14; 97C4-5, Bloomington stock number 5440). The identity of the gene was determined by non-complementation with the P-element allele *lnk^d07478^* (Bloomington stock number 19274) and subsequent sequencing of the *lnk* locus. Unless otherwise stated, a heteroallelic combination of *lnk* alleles (*lnk^4Q3^*/*lnk^6S2^*) was used to characterize the *lnk* phenotypes.

A 6 kb fragment spanning from the 3′ end of *CG17370* to the beginning of the first exon of *CG5913* was used as genomic rescue. The construct was inserted by means of ΦC31 mediated integration into a landing site on the second chromosome at 51D [40].

Constitutive active forms of InR (Bloomington stock number 8248) and of Dp110 (CAAX [41]) driven by *GMR-Gal4* were used for the epistasis analyses. For the generation of *chico*; *lnk* double mutant flies lacking one copy of *PTEN*, a deletion uncovering the *chico* and *PTEN* loci was used (*Df(2L)Exel6026*). To prove that the observed effect on the *chico*; *lnk* double mutants was caused by the loss of *PTEN*, *PTEN* was re-introduced by means of a genomic rescue construct. The *chico* alleles (*chico^1^* and *chico^2^*) have been described [8]. The heteroallelic combination *chico^1^*/*chico^2^* was used to compare *lnk* and *chico* mutants.

### Weight and Lipid Analyses

Flies of the respective genotypes were reared under identical conditions and collected 3 days after eclosion. They were dried at 95°C for 5 minutes and kept at room temperature for 3 days before weighing on a precision scale (Mettler Toledo MX5).

Three day-old flies were collected and weighted individually. Subsequent analysis of lipid content was performed as described [42].

### Clonal Analysis

Clones of *lnk* mutant cells were induced at 24–36 hours after egg deposition (AED) by heat shocking larvae of the genotype *y*, *w*, *hs-flp/y*, *w*; *FRT82*, *w+/FRT82*, *lnk^4Q3^* for 1 hour at 37°C. Fixation and tangential sections of the adult eyes were performed as described [43]. For the generation of mutant clones in the wing disc, animals of the genotype *y*, *w*, *hs-flp/y*, *w*; *FRT82*, *Ubi-GFP/FRT82*, *lnk^4Q3^* were exposed to a 5 minute heat shock at 37°C at 54–56 hours AED. Larvae were dissected 48 hours later, fixed in 4% paraformaldehyde on ice for 1 hour, and incubated in PBS containing DAPI (1∶2000) for 10 minutes. Discs were dissected and mounted in Vectashield Mounting Medium. Pictures were taken using a Leica SP2 confocal laser scanning microscope.

The quantification of the mutant clones was performed by comparing the size of the area occupied by mutant versus wild-type (pigmented) photoreceptor cells R6 using Photoshop CS2. In the wing discs, the numbers of nuclei within mutant and wild-type clones were counted and the areas were measured using Photoshop CS2.

### tGPH Localization and Ovarian Phenotypes

Larvae of the genotype *y*, *w*; *tGPH/+*; *FRT 82*, *w^+^/FRT82*, *lnk^4Q3^* were heat shocked 6–8 hours AED for 1 hour at 37°C, collected at wandering stage, fixed for 1 hour at room temperature in 8% paraformaldehyde and stained with DAPI (1∶10000 in PBS) for 20 minutes. Fat bodies were dissected and mounted in Vectashield Mounting Medium. Pictures were taken using a Leica SP2 confocal laser scanning microscope (Figure 4A–4D) and a Zeiss ApoTome microscope (Figure 4E–4E″), respectively.

Ovaries were dissected from 3 day-old wild-type, *lnk^4Q3^*/*lnk^6S2^* and *chico^1^*/*chico^2^* flies, respectively, and subsequently incubated in PBS containing DAPI (1∶2000) for 10 minutes. Thereafter, ovarioles were mounted in Vectashield Mounting Medium and pictures were taken using a Leica SP2 confocal laser scanning microscope.

### Western Blotting

Third instar larvae (10 mg of each genotype) were collected, briefly washed in PBS, transferred to 1.5 ml Eppendorf tubes and flash-frozen in liquid nitrogen. Larvae were homogenized in 75 µl of extraction buffer [44]. After 15 minutes incubation at 4°C and centrifugation at 12000 g for 15 minutes, protein concentrations were determined using the RC DC Protein Assay (Bio-Rad).

For the Western blots, 30 µg of protein samples were loaded, blotted and detected with the following antibodies: rabbit anti-Akt (Cell Signaling #9272, diluted at 1∶1000), rabbit anti-phospho-Drosophila Akt (Ser 505) (Cell Signaling #4054, diluted at 1∶500), and mouse anti-Actin (Sigma A5316, diluted at 1∶10000). HRP-conjugated secondary antibodies (Jackson ImmunoResearch) were diluted at 1∶10000. Signals were detected using ECL Western blotting detection reagents (Amersham Biosciences).

## Supporting Information

Figure S1 Structure-function analysis of Lnk. Tyrosines predicted to be phosphorylation targets within binding motifs for the SH2 domain of Drk and for Cbl, respectively, were specifically mutated to phenylalanine (see Figure 1D). Genomic rescue constructs carrying the respective mutations were introduced into a homozygous *lnk* mutant background. All mutations were able to complement the loss of *lnk* function with respect to size and weight. (A) Y-F mutation in the Cbl binding motif, (B) mutation in YXN90, (C) mutation in YXN576. Significant changes relative to the control (*p*≤0.01, Student's t-test, n = 20) are marked by double asterisks; error bars represent the standard deviation.
(0.33 MB TIF)

Figure S2 Homology within PH and SH2 domains. Alignment of the PH domain (A) and SH2 domain (B) sequences of *Drosophila* Lnk with the respective sequences of the human homologs. Sequence identity is marked by black boxes and similarity by grey boxes.
(0.64 MB TIF)
