# Supplementary figures and images for: The Drosophila SH2B Family Adaptor Lnk Acts in Parallel to Chico in the Insulin Signaling Pathway
Source: PLoS Genet. 2009 Aug 14;5(8):e1000596. doi: 10.1371/journal.pgen.1000596 (PMC2716533; doi:10.1371/journal.pgen.1000596)

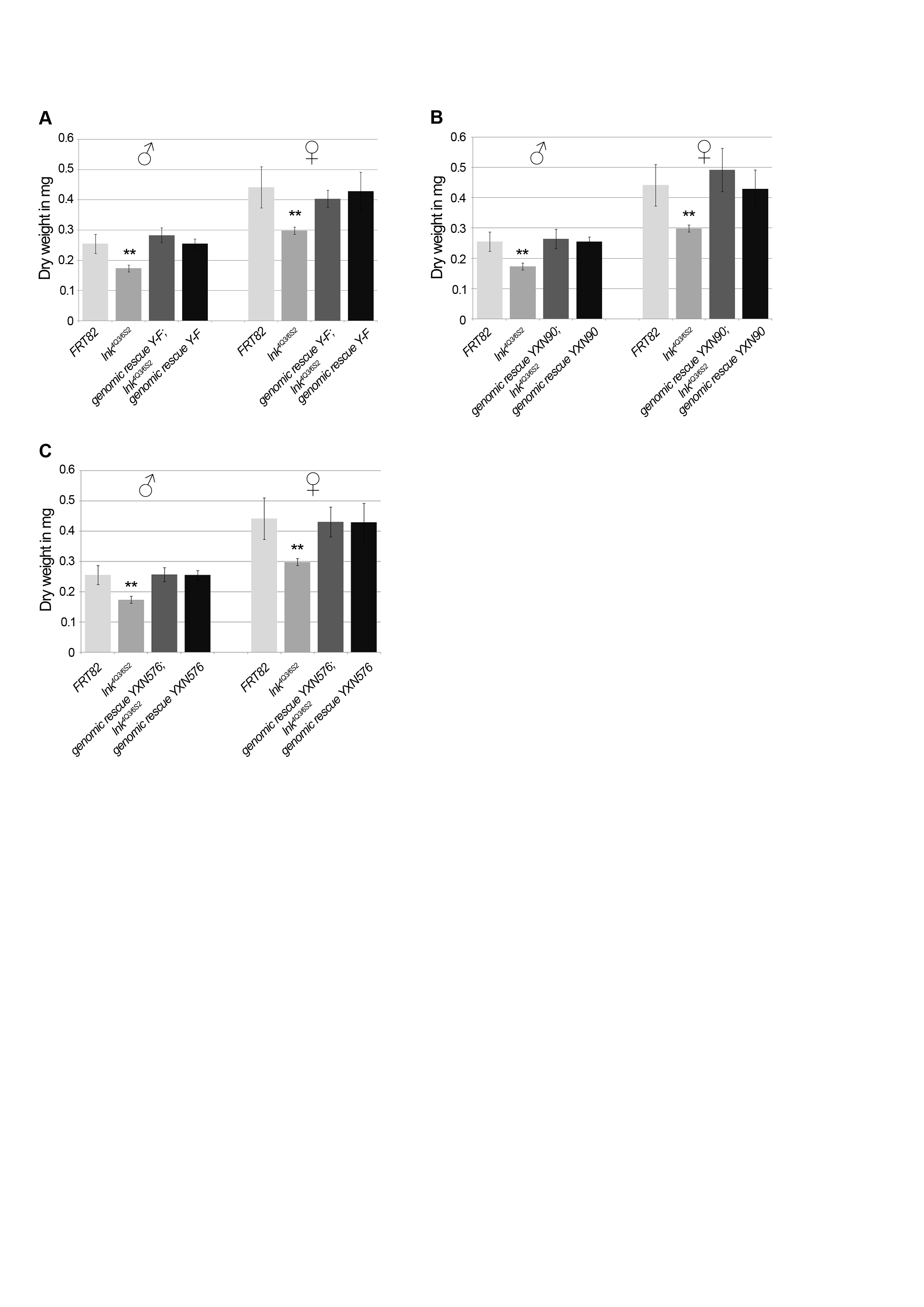

Supplement: Figure S1 — Structure-function analysis of Lnk. Tyrosines predicted to be phosphorylation targets within binding motifs for the SH2 domain of Drk and for Cbl, respectively, were specifically mutated to phenylalanine (see Figure 1D). Genomic rescue constructs carrying the respective mutations were introduced into a homozygous lnk mutant background. All mutations were able to complement the loss of lnk function with respect to size and weight. (A) Y-F mutation in the Cbl binding motif, (B) mutation in YXN90, (C) mutation in YXN576. Significant changes relative to the control (p≤0.01, Student's t-test, n = 20) are marked by double asterisks; error bars represent the standard deviation. (0.33 MB TIF) [file pgen.1000596.s001.tif]

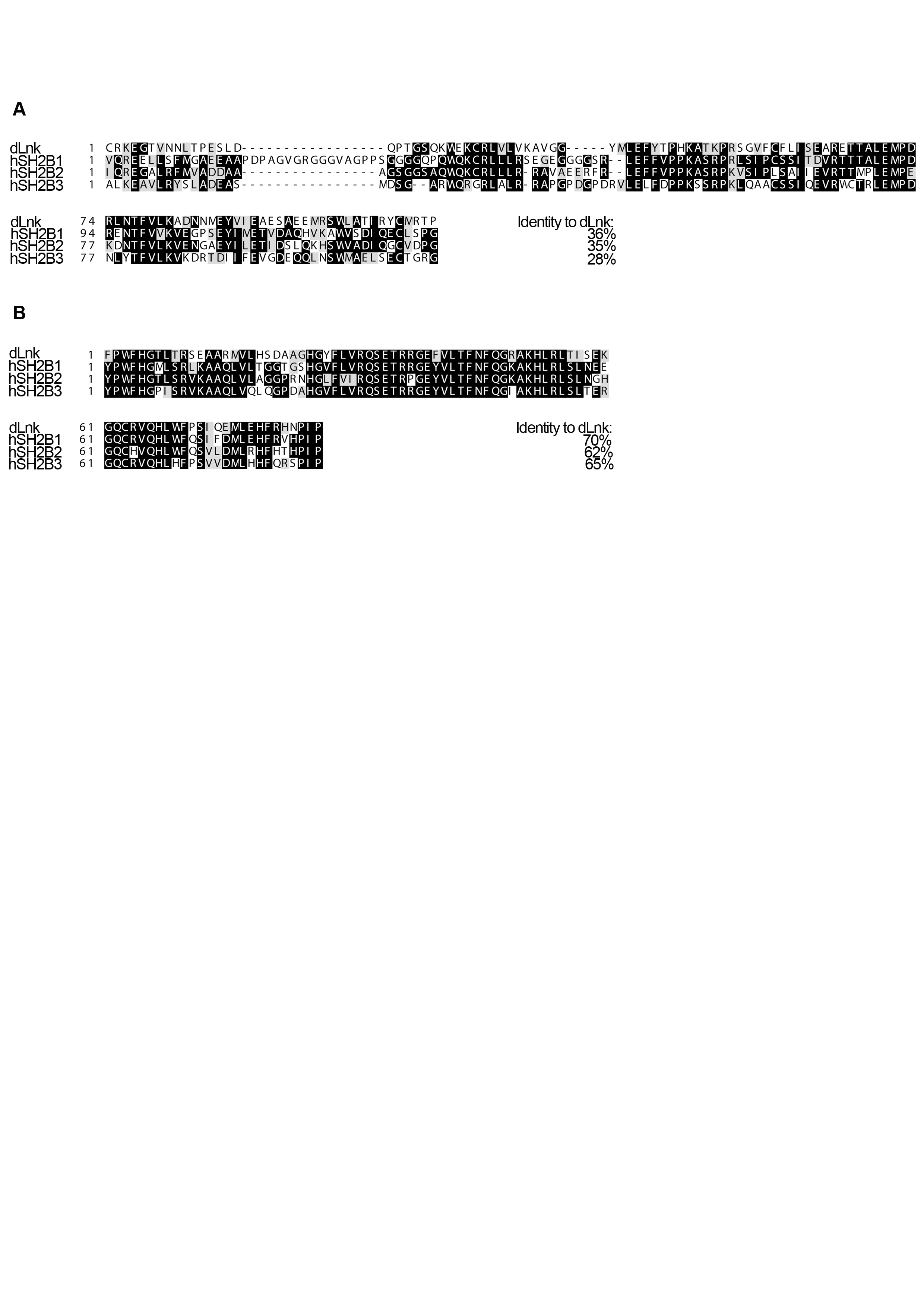

Supplement: Figure S2 — Homology within PH and SH2 domains. Alignment of the PH domain (A) and SH2 domain (B) sequences of Drosophila Lnk with the respective sequences of the human homologs. Sequence identity is marked by black boxes and similarity by grey boxes. (0.64 MB TIF) [file pgen.1000596.s002.tif]
